# Ectodomain shedding of Limbic System-Associated Membrane Protein (LSAMP) by ADAM Metallopeptidases promotes neurite outgrowth in DRG neurons

**DOI:** 10.1038/s41598-017-08315-0

**Published:** 2017-08-11

**Authors:** Ricardo L. Sanz, Gino B. Ferraro, Marie-Pier Girouard, Alyson E. Fournier

**Affiliations:** Montreal Neurological Institute, Department of Neurology and Neurosurgery, Montreal, H3A 2B4 Canada

## Abstract

IgLONs are members of the immunoglobulin superfamily of cell adhesion proteins implicated in the process of neuronal outgrowth, cell adhesion and subdomain target recognition. IgLONs form homophilic and heterophilic complexes on the cell surface that repress or promote growth depending on the neuronal population, the developmental stage and surface repertoire of IgLON family members. In the present study, we identified a metalloproteinase-dependent mechanism necessary to promote growth in embryonic dorsal root ganglion cells (DRGs). Treatment of embryonic DRG neurons with pan-metalloproteinase inhibitors, tissue inhibitor of metalloproteinase-3, or an inhibitor of ADAM Metallopeptidase Domain 10 (ADAM10) reduces outgrowth from DRG neurons indicating that metalloproteinase activity is important for outgrowth. The IgLON family members Neurotrimin (NTM) and Limbic System-Associated Membrane Protein (LSAMP) were identified as ADAM10 substrates that are shed from the cell surface of DRG neurons. Overexpression of LSAMP and NTM suppresses outgrowth from DRG neurons. Furthermore, LSAMP loss of function decreases the outgrowth sensitivity to an ADAM10 inhibitor. Together our findings support a role for ADAM-dependent shedding of cell surface LSAMP in promoting outgrowth from DRG neurons.

## Introduction

During development, membrane associated and soluble proteins direct the extent and trajectory of growing projections. The immunoglobulin superfamily of cell adhesion molecules (IgSF) is an important regulator of neurite outgrowth. Clustering of IgSF proteins across the cell surface increases adhesive interactions and stabilizes protein tyrosine kinase receptors, resulting in growth-promoting or repressive signalling events^1–3^. Members of the IgLON subfamily of IgSF-cell adhesion proteins are the earliest and most abundant glycosylphosphatidylinositol (GPI)-anchored proteins expressed in the nervous system. Adhesive interactions between IgLON family members across the cell surface or between adjacent cells regulates neuronal growth, cell adhesion and subdomain target recognition^4–6^. IgLON family members, Neurotrimin (NTM), Opioid binding cell adhesion molecule (OBCAM), Limbic system-associated membrane protein (LSAMP) and Neuronal growth regulator 1 (NEGR1), are characterized by three Ig-like domains that are attached to the cell membrane through a GPI-anchor moiety.

On the cell surface, IgLONs exist as dimeric structures capable of repressing or promoting growth, depending on the neuronal population, the developmental stage and surface repertoire of IgLON family members^7–11^. We previously reported IgLONs to be shed from the cell surface via a post-translational mechanism termed ectodomain shedding. Surface proteolysis is mediated by members of the metzincin family of metalloproteinases including matrix metalloproteinases (MMPs) and a disintegrin and metalloproteinases (ADAMs). Genetic and pharmacological manipulations have demonstrated a requirement for metalloproteinases for the guidance and growth of axons during development^12, 13^. Metalloproteinases control levels of cell surface proteins, activate signaling upon ligand binding and release biologically active, or dominant negative protein fragments. By regulating levels of cell surface receptors, axons can switch responsiveness to environmental cues and modulate their outgrowth response. In aged cortical neurons, metalloproteinase-dependent shedding of IgLON family members generates a permissive substrate for neurite outgrowth^14^. However, whether IgLON proteolysis is implicated in the growth of other neuronal populations remains unclear.

In the present study, we evaluated the role of IgLON shedding in the growth of dorsal root ganglion cells (DRGs). Dorsal root ganglia are formed by the cell bodies of sensory neurons, which project one process towards the dorsal horn of the spinal cord and another process to peripheral tissues to relay sensory information from the periphery into the central nervous system. Here, we identify a metalloproteinase-dependent mechanism for neurite outgrowth in embryonic DRGs. Treatment of embryonic DRG neurons with pan-metalloproteinase inhibitor and an ADAM10 inhibitor reduces outgrowth from DRG neurons. We identify IgLON family members, NTM and LSAMP, as two metalloproteinase substrates that are shed from the cell surface of DRG neurons. Overexpression of LSAMP and NTM represses outgrowth from DRG neurons. Furthermore, LSAMP downregulation attenuates the sensitivity of DRGs to an ADAM10 inhibitor. We thus define a role for metalloproteinase-dependent shedding of surface LSAMP in releasing a brake on neurite outgrowth from DRG neurons.

## Results

### Pan-metalloproteinase inhibitors repress growth in embryonic DRG neurons

**Figure 1:**
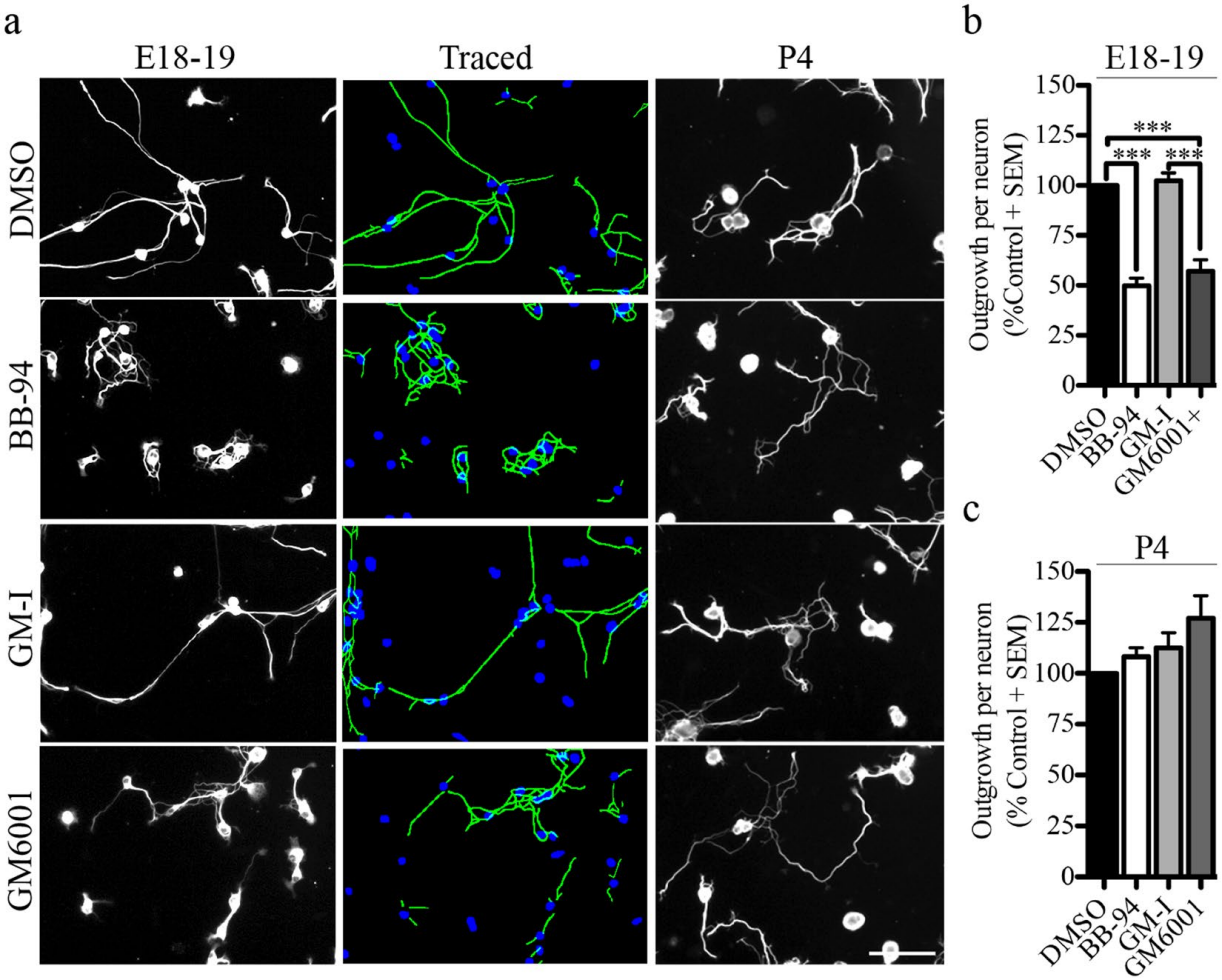
Pan-metalloproteinase inhibitors repress neurite outgrowth in embryonic rat DRG neurons. (**a**) Dorsal root ganglion cells from two developmental stages (E18-19 and P4) seeded on Poly-L-lysine (PLL, 100 μg/mL) coated 96-well plate and treated with a vehicle control (DMSO), metalloproteinase inhibitors (BB-94 and GM6001), and a GM6001-Inactive (GM-I) control for 24 hrs. Neuronal projections were visualized with βIII tubulin and representative images of traced neurites for the outgrowth quantification are presented. (**b**,**c**) Outgrowth quantification from E18-19 (**b**) and P4 (**c**) DRG neurons. Outgrowth was normalized to DMSO control for each experiment. N = 4 from independent cultures. Data shown as mean + S.E.M. ***p < 0.001 by one-way ANOVA, followed by Bonferroni post hoc test. Scale bar, 100 μm.

**Figure 2:**
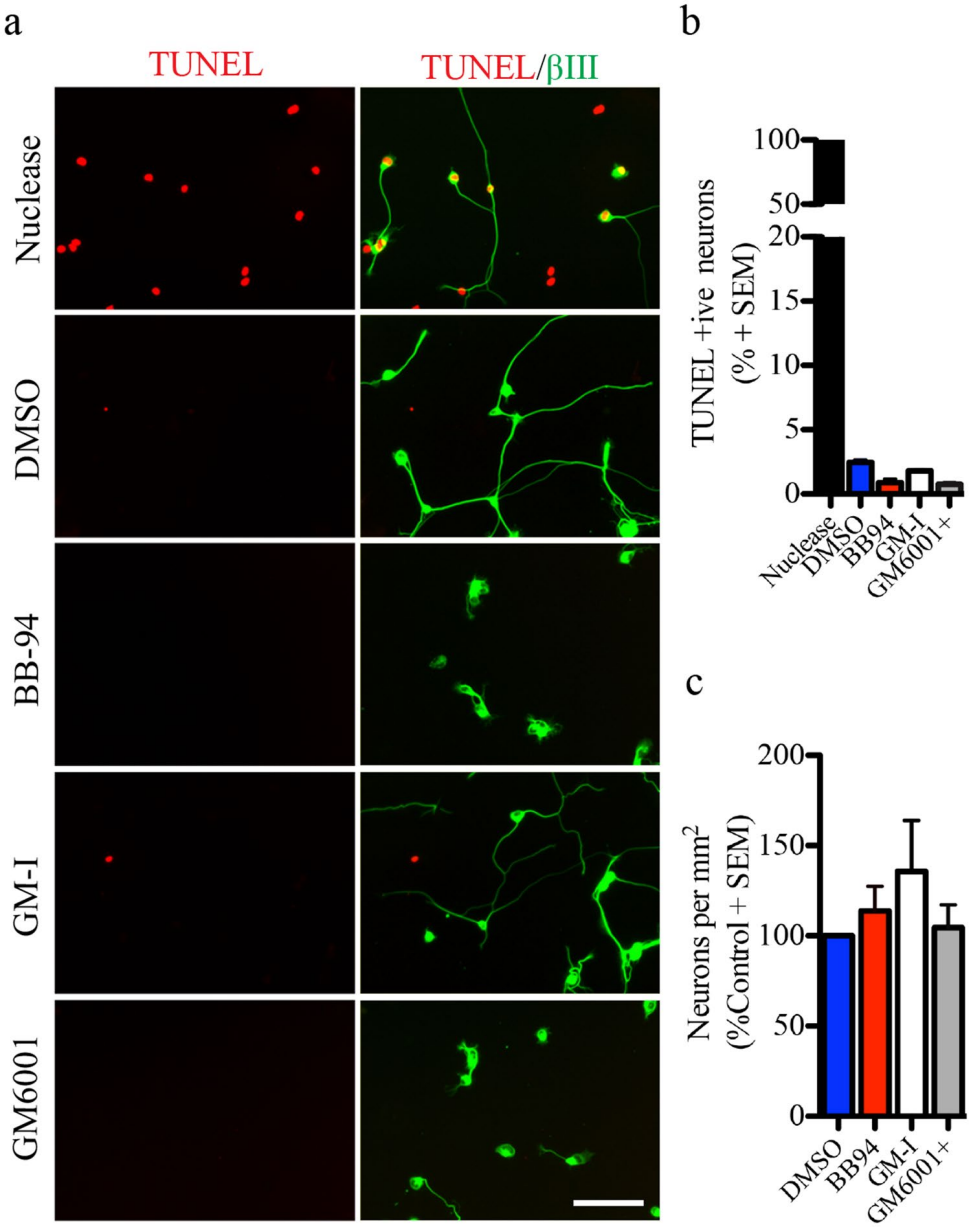
Pan-metalloproteinase inhibitors do not affect adhesion or cell death of DRG neurons. (**a**) A TUNEL assay was performed in embryonic DRG neurons (E18-19) seeded on a PLL substrate and treated with a vehicle control (DMSO), metalloproteinase inhibitors (BB-94 and GM6001) or GM-I. Neurons were stained with anti-βIII tubulin, Hoechst and TUNEL. As a positive control for the TUNEL assay, neurons were pretreated with a DNA nuclease. (**b**,**c**) Total number of apoptotic neurons (**b**) and total neurons (**c**) present after indicated treatments. For TUNEL, the data are shown as a percentage of TUNEL-positive neurons. For total number of neurons, data have been normalized to control DMSO. N = 4 from independent cultures. Data are shown as mean + S.E.M. Scale bar, 100 μm.

In the present study, we evaluated the role of metalloproteinases in the neurite outgrowth of sensory dorsal root ganglion neurons. We examined outgrowth of DRGs harvested from embryonic (E18-19) and postnatal (P4-6) rats and seeded on a poly-L-lysine (PLL) substrate. Neurons were exposed to BB-94 and GM6001, synthetic hydroxamate-based inhibitors that target both matrix metalloproteinases and adamalysins, and the catalytically inactive GM6001 (GM-I) as a negative control. After 24 hrs, metalloproteinase inhibition resulted in a 50% reduction in the growth of embryonic DRG neurons (Fig. 1a,b). The metalloproteinase inhibitors had no effect on the outgrowth of early postnatal DRG neurons revealing a developmental switch in the outgrowth promoting effect of metalloproteinases (Fig. 1c). To determine whether pan-metalloproteinase inhibitors indirectly repress growth through a decrease in cell viability or cell adhesion, we quantified the number of neurons and apoptotic neurons following treatment with BB-94 and GM6001. Metalloproteinase inhibitors did not affect the number of neurons adhering to the substrate nor the number of apoptotic neurons stained with TUNEL TMR-red (Fig. 2a–c). We conclude that metalloproteinases directly promote outgrowth from embryonic DRG neurons.

### ADAM protease activity is necessary to promote growth in embryonic DRG neurons

**Figure 3:**
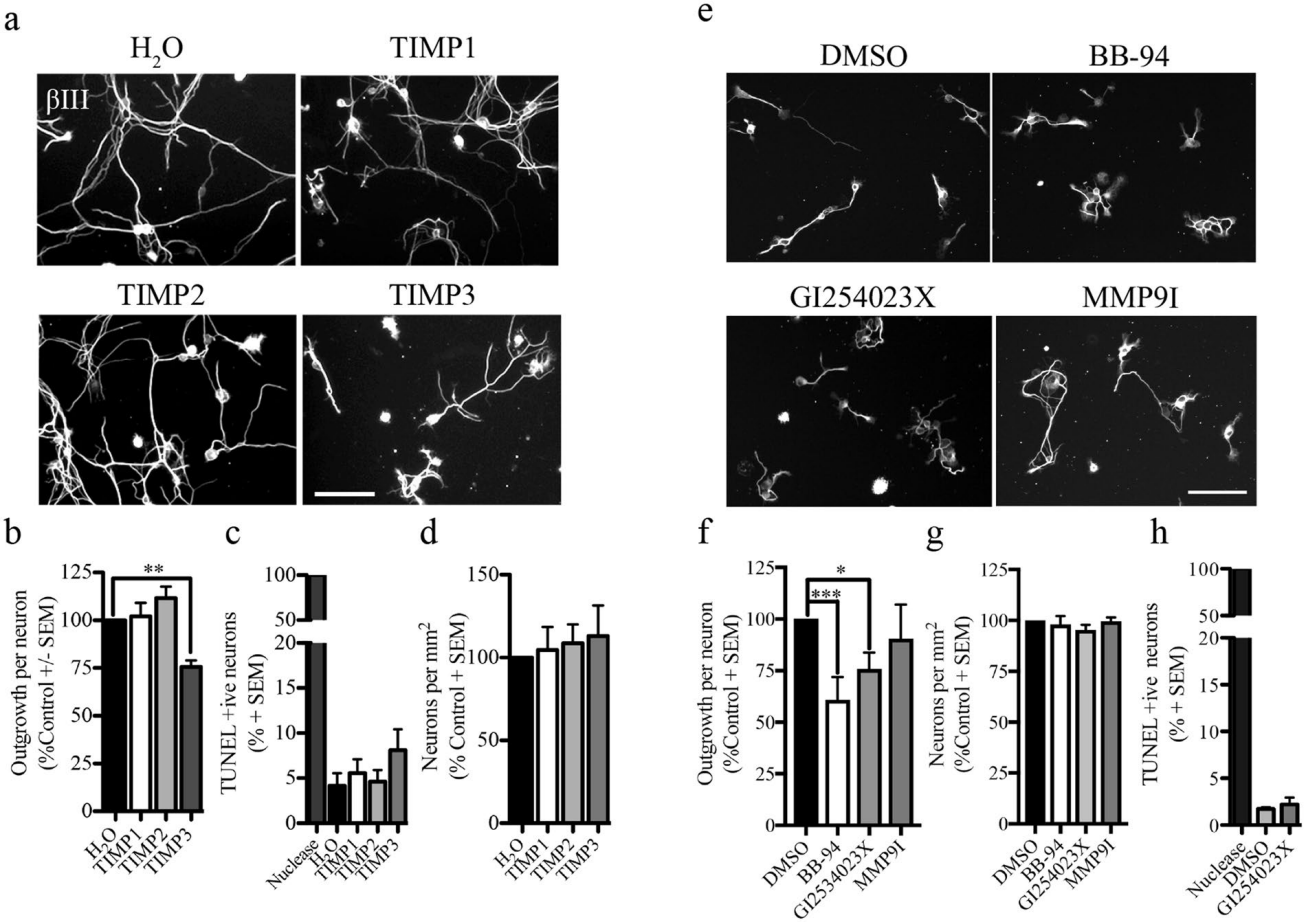
ADAM proteolytic activity is necessary for embryonic DRG growth. (**a**) Embryonic DRG neurons seeded on a PLL substrate and treated with control (H_2_O), or endogenous metalloproteinase inhibitors (TIMP1-3). Neuronal projections were visualized with βIII-tubulin staining. (**b**) Neurite outgrowth quantification of embryonic DRG neurons exposed to soluble recombinant TIMPs and control conditions. Data have been normalized to control condition. (**c**) TUNEL assay was performed in embryonic DRG neurons treated with TIMPs or H_2_O control. DNA nuclease was used as a positive control. Data are shown as a percentage of TUNEL-positive neurons. (**d**) Number of neurons present on the PLL substrate after treatment with soluble TIMPs and control condition. (**e**). Embryonic DRG neurons exposed to DMSO as a control, a selective ADAM10 inhibitor, GI254030X, BB-94 or a selective MMP9 inhibitor (MMP9I). (**f**–**h**) Quantification of neurite outgrowth (**f**), cell adhesion (**g**) and percentage TUNEL-positive neurons (**h**) following treatment with metalloproteinase inhibitors. For the TUNEL assay the data are shown as a percentage of TUNEL-positive neurons. For total number of neurons, data have been normalized to control DMSO or H_2_O. N = 3–4 from independent cultures. Data are shown as mean + S.E.M. *p < 0.05, **p < 0.01, ***P < 0.001by one-way ANOVA followed by Bonferroni post hoc test. Scale bar, 100 μm.

To investigate the specific metalloproteinase responsible for promoting embryonic DRG growth, we performed outgrowth assays in the presence of endogenous zinc-metalloproteinase inhibitors. Tissue inhibitors of metalloproteinases (TIMPs) are a family of endogenous metalloproteinase inhibitors that differ in their substrate selectivity. TIMP1 represses the activity of secreted MMPs, TIMP2 represses all matrix metalloproteinases and TIMP3 represses metalloproteinases and some ADAM family members. Exposure to recombinant TIMP3, but not TIMP1 nor TIMP2 resulted in a significant reduction in DRG outgrowth potentially implicating an ADAM family member (Fig. 3a,b). Similar to pan-metalloproteinase inhibitors, TIMP3 had no effect in cell adhesion or in cell viability, (Fig. 3c,d).

Among the few studies reporting phenotypes of mice lacking individual ADAM family members, ADAM10 deficiency results in embryonic lethality with pronounced defects in neuronal and cardiovascular systems. ADAM10 is essential for the establishment of the cerebral cortex, the guidance of proprioceptive neurons during development and the processing of different adhesion proteins present on the cell surface^15, 16^. Furthermore, ADAM10 is prominently expressed in embryonic DRG neurons, and relatively absent in aged DRGs^17^. To examine the effect﻿ o﻿f ADAM10 proteolytic activity in the growth of DRG projections, we performed outgrowth experiments in embryonic DRG neurons treated with GI254023X, a selective ADAM10 inhibitor^18–20^. Neurons were also treated with an inhibitor of MMP-9 (MMP9I), another TIMP3 substrate to assess the specificity of the effect. GI254023X, but not MMP-9I, repressed DRG outgrowth without affecting survival and adhesion, and this effect was similar to the effects observed with the pan-metalloproteinase inhibitor BB-94 (Fig. 3e–h). This result is consistent with reports of ADAM10 inhibitors suppressing axon growth in DRG-Schwann cell co-cultures^17^. We conclude that an ADAM metalloprotease, most-likely ADAM10, is important for the growth of embryonic DRG neurons.

### NTM and LSAMP are shed from the surface of embryonic DRG neurons by ADAM10

**Figure 4:**
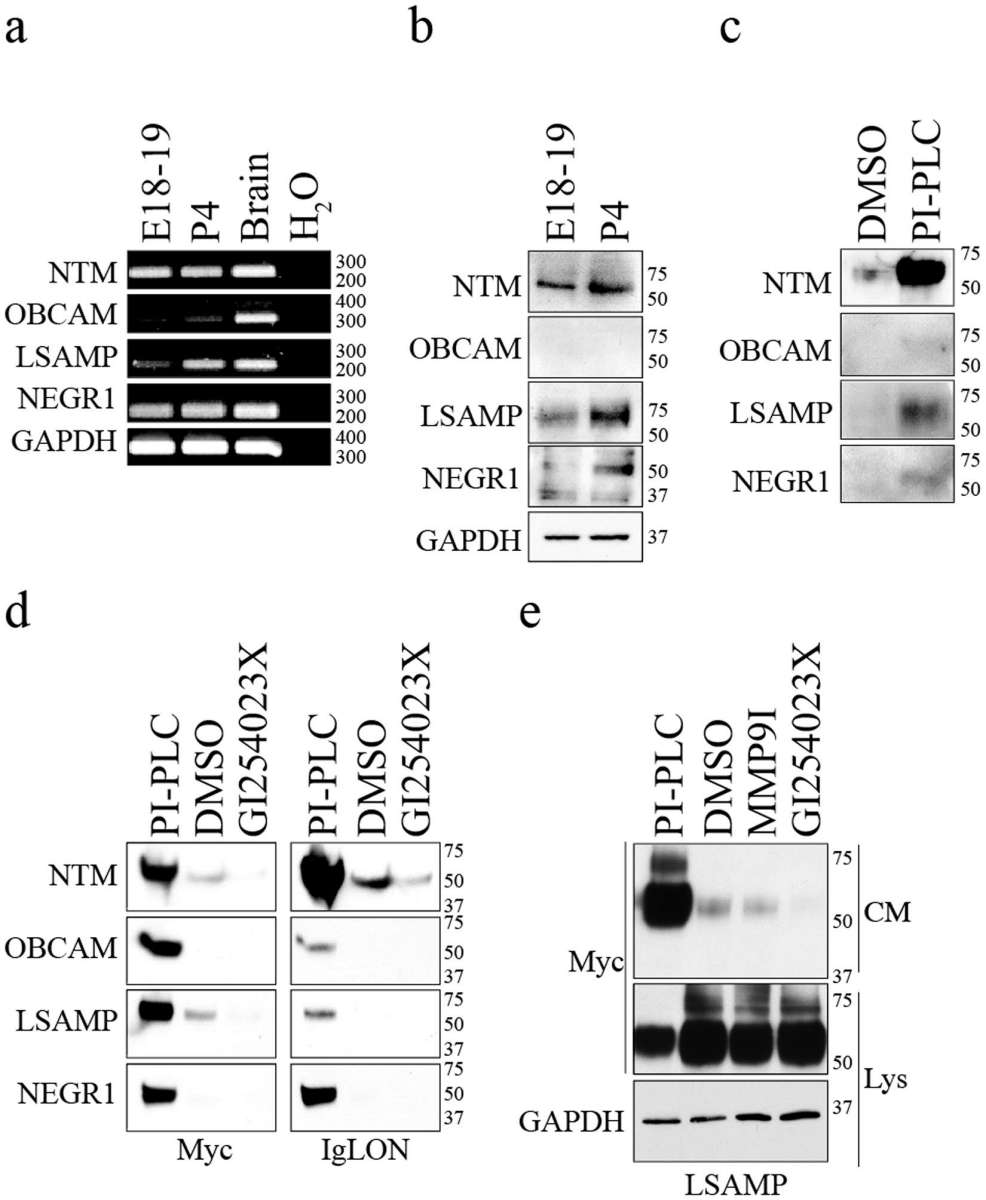
NTM and LSAMP are expressed and shed from the surface of embryonic DRG neurons by ADAM10. (**a**) Reverse transcription PCR (RT-PCR) was performed in DRG neurons from two developmental stages (E18-19 and P4). Brain RNA was used as a positive control and H_2_O as a negative control. GAPDH RNA expression was analyzed as a loading control. (**b**) Lysates from embryonic (E18) and postnatal (P4) DRG neurons analyzed by Western Blot to assess the expression of endogenous IgLON proteins. GAPDH was analyzed as a loading control. (**c**) Conditioned media from membrane-extracts from (E18-19) DRG exposed to PI-PLC, as a positive control. Commercially available IgLON antibodies were used to detect cleaved IgLON fragments in the media. (**d**) The potency of commercially available IgLON antibodies was assessed by Western Blot. Conditioned media from dissociated embryonic (E18-19) DRG neurons expressing Myc-tagged IgLON constructs were probed with commercial anti-IgLON antibodies and anti-Myc antibodies. (**e**) Western Blot analysis of conditioned media from DRG neurons transduced with myc-tagged LSAMP and treated with the ADAM10 inhibitor GI254023X or the MMP9 inhibitor MMP9I. Western blots were cropped and full length blots are provided in Supplementary Figure S1.

Previously, we identified IgLON family members as novel metalloproteinase substrates that promote growth in mature cortical neurons when shed from the cell surface^10^. We therefore asked if IgLON family members are important substrates for ADAM-dependent outgrowth of DRG neurons. We assessed expression of IgLON RNA from embryonic (E18-19) and postnatal (P4-6) DRGs by reverse-transcription polymerase chain reaction. NTM, OBCAM, LSAMP and NEGR1 were detected at both developmental stages (Fig. 4a). At the protein level, NTM, LSAMP and NEGR1 protein was present in embryonic and postnatal DRG lysates, while OBCAM was undetectable (Figs 4b, S1a). LSAMP RNA and protein exhibited a developmental upregulation at postnatal day 4, whereas NTM, OBCAM and NEGR1 expression remained stable (Fig. 4a,b).

To examine the processing of IgLON family members, conditioned media from DRG membrane extracts was analyzed for deposition of IgLON shed fragments. Membranes were treated with PI-PLC to cleave all GPI-anchored proteins as a positive control. Shedding of all IgLON family members was detectable following treatment with PI-PLC (Figs 4c, S1b). Under baseline conditions (DMSO treated), NTM was clearly shed from the cell surface, while LSAMP was weakly detected. IgLON bands detected in the PI-PLC condition raised the possibility that all IgLON family members are shed endogenously under baseline conditions but are below the level of detection for these antibodies. To explore this issue, we analyzed conditioned media from dissociated DRGs infected with Myc-tagged IgLONs. All four IgLON family members were detected with an anti-Myc antibody in conditioned media following PI-PLC treatment (Figs 4d, S1c and S1d). Consistent with the endogenous shedding result, NTM and LSAMP were released from untreated membranes (DMSO) and this release was reduced upon treatment with the ADAM10 inhibitor GI254023X (Fig. 4d). The selectivity of ADAM10 effect is further supported by the failure of an MMP9 inhibitor to suppress LSAMP shedding (Figs 4e, S1e). Shedding of OBCAM and NEGR1 was undetectable unless membranes were treated with PI-PLC (Fig. 4d). When membranes were probed with anti-IgLON antibodies, we found that the anti-NTM and anti-NEGR1 antibodies were robust whereas anti-OBCAM and anti-LSAMP antibodies weakly detected their target (Fig. 4d). We conclude that NTM and LSAMP are most-likely shed from the surface of embryonic DRG neurons in an ADAM10-dependent manner.

### LSAMP represses growth in embryonic DRG neurons

**Figure 5:**
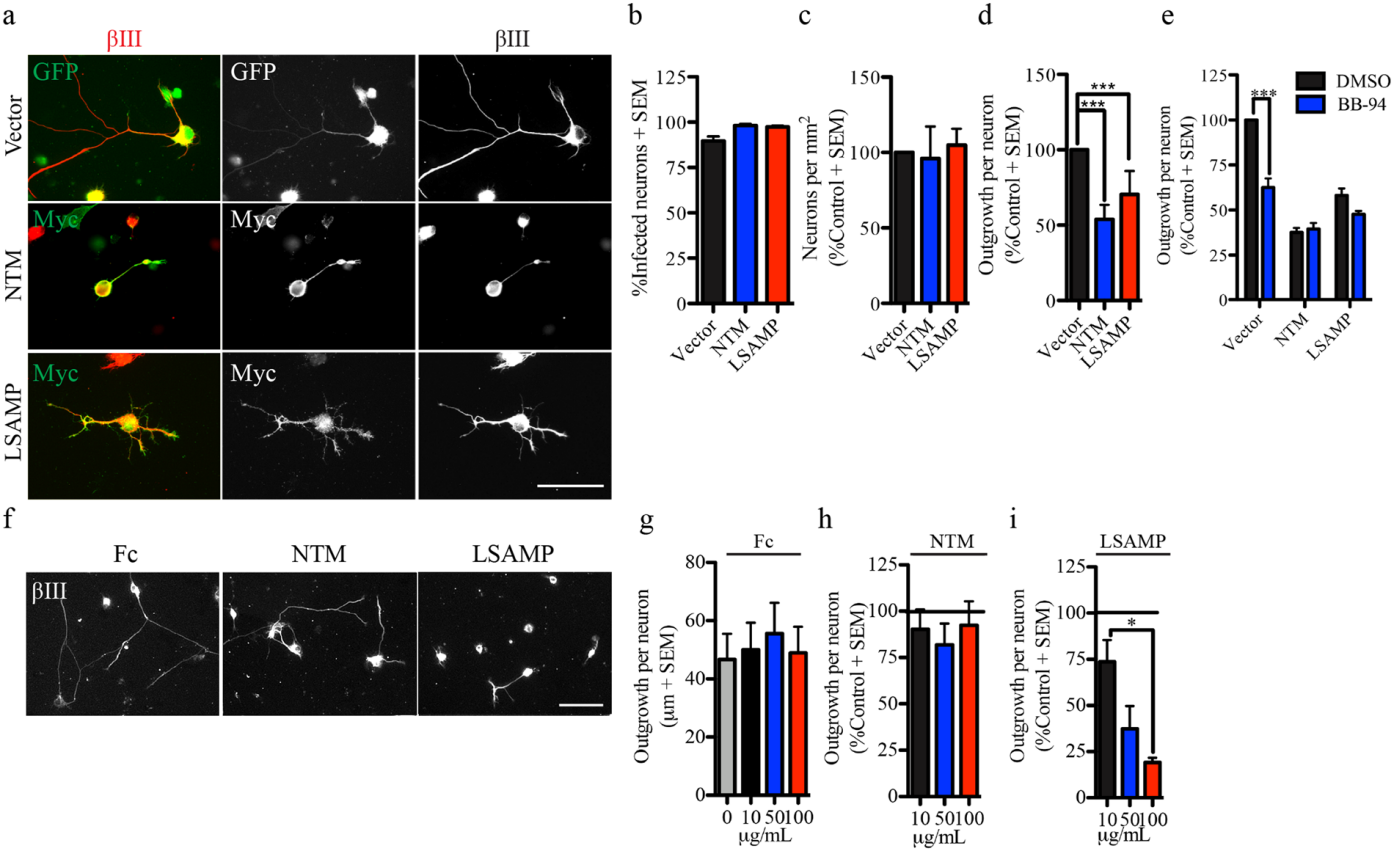
Ectopic surface NTM and LSAMP repress neurite outgrowth in embryonic DRG neurons. (**a**) Embryonic (E18-19) DRG neurons seeded on a PLL substrate and expressing HSV-GFP or HSV-Myc-tagged IgLON constructs. Surface Myc-IgLON expression was detected with anti-Myc antibody in the absence of permeabilization. (**b**–**e**) Quantification of the number of infected neurons (**b**), total number of seeded neurons (**c**) and neurite outgrowth (**d**,**e**) from E18-19 DRG neurons expressing GFP (vector) or Myc-IgLON proteins in the presence or absence of BB-94. Data were normalized to control HSV-GFP. N = 3–6 from independent cultures. (**f**). βIII tubulin stained E18-19 DRG neurons plated on immobilized Fc or FC-IgLON substrates. (**g**–**i**) Quantification of outgrowth from embryonic DRG neurons seeded on immobilized Fc-IgLON substrates (10, 50 and 100 μg/mL). Data were normalized to control Fc substrate. N = 4 from independent cultures. Data are shown as mean + S.E.M. *p < 0.05, **p < 0.01, ***P < 0.001 by one-way ANOVA followed by Bonferroni post hoc test. Scale bar, 50 μm.

We next asked if ADAM10-dependent outgrowth inhibition could be ascribed to stabilization of inhibitory NTM or LSAMP expression on the cell surface. To test this scenario, we asked if cell surface IgLON expression suppresses DRG outgrowth. NTM and LSAMP were introduced into embryonic DRG neurons and cell surface expression was verified by immunofluorescence analysis of transduced neurons in the absence of permeabilization (Fig. 5a,b). Overexpression of both NTM and LSAMP restricted outgrowth of DRG neurons without affecting their ability to adhere to the substrate (Fig. 5c,d). Furthermore, neurite outgrowth from neurons overexpressing NTM or LSAMP was not further inhibited by BB-94 (Fig. 5e). This supports the idea that metalloproteinase inhibitors may suppress DRG outgrowth by stabilizing inhibitory IgLON expression on the cell surface. When DRG neurons were plated on IgLON substrates, we found that LSAMP but not NTM had an inhibitory effect on neurite outgrowth (Fig. 5f–i). Thus, the shedding of IgLON proteins from the surface of DRG neurons does not generate a permissive substrate for neurite outgrowth, as we have previously shown to be the case for cortical neurons^14^.

### LSAMP loss of function desensitizes DRG neurons to an ADAM10 inhibitor

**Figure 6:**
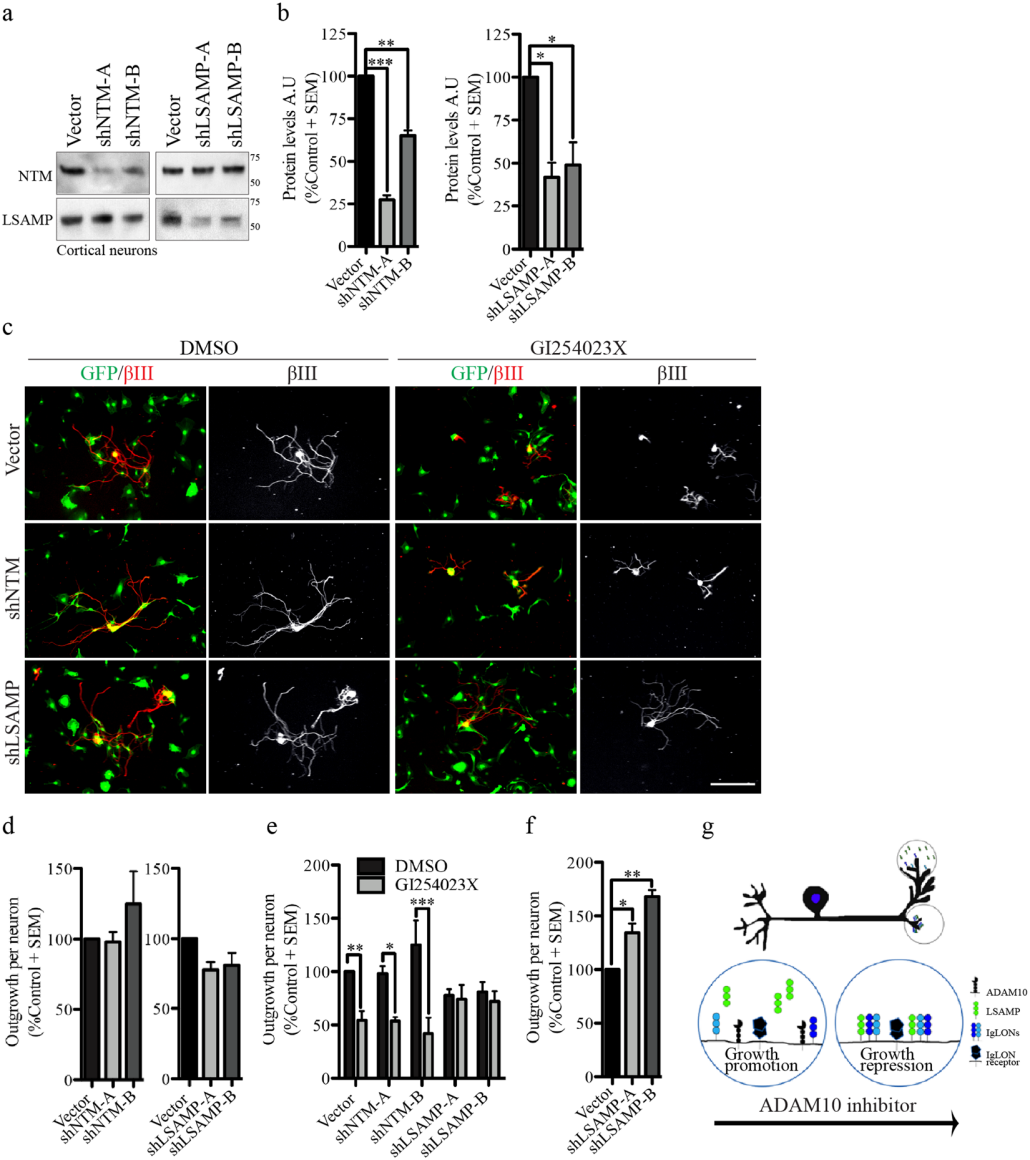
LSAMP loss of function desensitizes DRG neurons to an ADAM10 inhibitor. (**a**) Western Blot of conditioned media from embryonic cortical neurons expressing control lentivirus (vector), NTM-shRNAmir (shNTM-A and shNTM-B) or LSAMP-shRNAmir (shLSAMP-A and shLSAMP-B) for 8 days and treated with PI-PLC for 1 hr. Full length Western blots provided in Fig. S2a. (**b**) Densitometry analysis of NTM and LSAMP proteins present in the media after PI-PLC treatment. (**c**) βIII tubulin staining of embryonic DRG (E18-19) neurons infected with control GFP-lentivirus, NTM-shRNAmir or LSAMP-shRNAmir for 8 days, reseeded on PLL coated plates and exposed to DMSO or GI254023X for 24 hrs. (**d**,**e**) Quantification of neurite outgrowth of reseeded embryonic DRG neurons expressing vector GFP, NTM-shRNAmir or LSAMP-shRNAmir (**d**) and exposed to DMSO or GI254023X (**e**). (**f**) Quantification of neurite outgrowth from reseeded postnatal DRG neurons expressing vector GFP or LSAMP-shRNAmir. N = 3 from independent cultures. (**g**) Schematic representation of IgLON shedding by ADAM10 activity during embryonic development and the effects of ADAM10 inhibition in neurite outgrowth. Data shown as mean + S.E.M. *p < 0.05, **p < 0.01, ***p < 0.001 by one-way ANOVA, followed by Bonferroni post hoc test. Scale bar, 100 μm.

To determine if IgLON expression is required for ADAM10-dependent suppression of neurite outgrowth, we performed IgLON knockdown experiments and assessed neurite outgrowth in the presence and absence of GI254023X. NTM and LSAMP shRNA with a microRNA stem (shRNAmir) were introduced in embryonic DRG neurons. The efficiency of NTM and LSAMP knockdown was validated by western blotting (Figs 6a,b, S2a). After 8 days, DRGs were reseeded and allowed to grow for 24 hrs. Somewhat unexpectedly, knockdown of NTM or LSAMP did not significantly enhance neurite outgrowth on E18-19 DRG neurons (Fig. 6c,d). Coupled with the ability of NTM and LSAMP overexpression to suppress growth (Fig. 5), we reasoned that endogenous levels of NTM and LSAMP in embryonic DRG neurons are insufficient to suppress growth and that inhibition of ADAM10 may accumulate sufficient IgLONs on the cell surface to suppress outgrowth. Consistent with this model, knockdown of LSAMP, but not NTM, rendered neurons insensitive to the inhibitory effects of the ADAM10 inhibitor, GI254023X (Fig. 6e). This demonstrates that GI254023X requires LSAMP to suppress neurite outgrowth. In postnatal (P4-6) DRG neurons that express higher levels of LSAMP, knockdown of LSAMP was sufficient to induceneurite outgrowth (Fig. 6f). Together, this supports a model whereby ADAM10 proteolytic cleavage of LSAMP relieves a brake on neurite outgrowth inhibition that facilitates axon extension of developing DRG neurons (Fig. 6g).

## Discussion

Ectodomain shedding is an important post-translational mechanism that can destabilize surface receptor complexes, desensitize cells to ligands, and generate biologically active fragments. In the present study, we identified a member of the ADAM metalloproteinase subfamily, ADAM10, as an important mediator of neurite outgrowth in sensory DRG neurons (Fig. 1). Other groups have reported that ADAM10 promotes growth of retinal ganglion cells and cerebellar ganglion cells by processing cell adhesion proteins N-Cadherin^21^ and L1^22^, respectively. Previously, we reported the ADAM family of metalloproteinases as potential mediators of IgLON processing^14^. NEGR1 processing in mature cortical neurons was recently described to promote growth and neuronal arborization^23^. Here, we characterized the IgLON family of cell adhesion proteins to be shed from the surface of embryonic DRGs by ADAM10 to facilitate neurite outgrowth. Through reverse transcription polymerase chain reaction and analysis of protein lysates from embryonic DRG ganglia, we identified two IgLON family members, NTM and LSAMP, robustly expressed in DRG neurons. In shedding experiment from transduced DRGs, only NTM and LSAMP fragments were strongly detected in the conditioned media and reduced in the presence of GI254023X. This may reflect limited stability for NEGR1 and OBCAM upon their release from the cell surface, or a limited affinity for ADAM10 proteolytic activity.

In a previous publication, we proposed two potential mechanisms for IgLON proteolysis in neurite outgrowth: IgLON shed fragments could provide a growth permissive substrate, or could relieve an outgrowth inhibitory signal from the cell surface^14^. In DRG neurons, our data supports the latter mechanism. This differs from the mechanism in cortical neurons where shed IgLON forms a growth permissive substrate. The outgrowth sensitivity to GI254023X results from enrichment of cell surface IgLONs that limits the extension of DRG neurons. Accordingly, NTM and LSAMP introduced in embryonic DRG neurons mimic the outgrowth inhibition with GI254023X, implying that accumulation of surface IgLONs represses neurite outgrowth. A hierarchy for IgLON interactions has previously been reported. In a cell-based ELISA assay, LSAMP expressing CHO cells exhibit the strongest binding with soluble NTM-Fc and OBCAM-Fc^11^, suggesting that LSAMP may have a unique capacity to form IgLON heterophilic complexes and stabilize IgLONs expressed on the neuronal surface to limit growth. In DRG neurons we find that immobilized LSAMP substrates, but not NTM, repress neurite outgrowth in a dose-dependent manner. Similarly, loss of surface LSAMP expression, but not NTM, desensitized DRG projections to GI254023X, supporting a mechanism where an outgrowth inhibitory signal is generated by the accumulation of LSAMP at the cell surface. Other groups have reported no outgrowth defect in E16 chick DRG neurons overexpressing NTM (CEPU-1), or seeded on immobilized IgLON substrates^24^. An interesting possibility is that the ratio of LSAMP expression to other IgLONs at this later stage of development may explain why IgLONs have disparate effects in different neuronal populations and at different stages of development.

In the nervous system, IgLONs are important mediators of axon patterning and fine connectivity. Anti-LSAMP antibodies have been reported to induce aberrant Timm-stained fibers throughout the CA3 region of the hippocampus^25^, and to significantly decrease the outgrowth of dopaminergic neurons towards the habenula^7^. Human genetic studies have found associations between LSAMP allelic polymorphisms and mood dysfunction, panic disorder, male suicide and schizophrenia^26, 27^, implying its importance in the formation of the brain circuitry. Furthermore, LSAMP deficient mice display hyperactivity in novel environments and reduced anxiety-like behaviors^28^. IgLONs are also characterized as tumor suppressor genes. NEGR1, OBCAM and LSAMP expressions are absent in clear renal cell carcinoma, epithelial ovarian cancer and in osteosarcomas, while restoring their expression decreases the rate of cell proliferation and tumor cell growth *in vivo* ^28, 29^. Based on our experiments, we hypothesize that IgLONs act as endogenous repressors of neurite outgrowth in DRG neurons and IgLON ectodomain shedding might relieve this outgrowth inhibition to closely regulate the extent of growth during outgrowth and target innervation. It is noteworthy that loss of LSAMP expression at embryonic stages did not affect the baseline DRG outgrowth (Fig. 6). We reason that at embryonic stages, the expression levels of endogenous LSAMP might be insufficient to repress neurite outgrowth, and only in the presence of metalloproteinase inhibitors, surface accumulation of unprocessed LSAMP might attain levels that limit growth. Accordingly, loss of LSAMP expression at postnatal stages promotes growth (Fig. 6), implying that LSAMP shedding might titer the extent of neurite outgrowth during DRG development.

It is intriguing that metalloproteinase inhibitors selectively inhibited the outgrowth of embryonic DRG neurons, while postnatal DRG neurons were unaffected (Fig. 1). The developmentally regulated response of DRG neurons is consistent with previous reports analyzing the expression of several ADAM family members in DRG-Schwann cell co-cultures^17^. During development, embryonic day 18–20 peripheral DRG projections have grown to the hindlimb and forelimb, while central DRG projections have entered the spinal cord without reaching their target destination. Proteolytic cleavage of IgLON family members could titrate DRG outgrowth and could have critical roles in subdomain targeting and synaptogenesis. In addition, ADAM10 activity decreases during the first week of neonatal life preceding the process of myelination. Interestingly, LSAMP has recently been described as a negative regulator of CNS myelination^30^. An interesting line of investigation would therefore be to examine the role of IgLON proteolysis and soluble Ecto-IgLON fragment in the proliferation, maturation and migration of Schwann cells.

## Methods

### Animals

Timed pregnant (embryonic day 18–19) female and postnatal (P4-6) Sprague Dawley rats were purchased from Charles River Laboratories. All animal care and use was in accordance with the McGill University guidelines and approved by the Montreal Neurological Institute Animal Care Committee.

### Antibodies

For immunofluorescence, the following antibodies were used: mouse and rabbit anti-tubulin βIII from Covance (1:1000) and mouse anti-Myc from Sigma-Aldrich (1:1000). Alexa-fluor secondary antibodies were purchased from Invitrogen Life Technologies (1:1000). For western blot analysis, the following antibodies were used: anti-NTM (1:100, R&D systems), anti-LSAMP (1:100, R&D systems), anti-OBCAM (1:100, Santa Cruz), anti-NEGR1 (1:100, Santa-Cruz), mouse and rabbit anti-Myc (1:500, Sigma-Aldrich) and anti-GAPDH (1:10000, Abcam). HRP-conjugated secondary antibodies were purchased from Jackson Immunoresearch.

### Plasmids, cloning and virus preparation

Full-length human cDNA sequences for NTM, and LSAMP (OpenBiosystems) were subcloned from a previously reported Psectag2B vector^14^ into an HSV-viral vector^31^. The following primers were used to generate IgLON viral constructs for overexpressionexperiments: NTM forward: 5′-GAATCTAGAATGGAGACAGACACACTC -3′; LSAMP forward: 5′- GAAGTCGACATGGAGACAGACACACTC-3′, NTM reverse: 5′- GAAGAGCTCTCAAAATTTGAGAAGCAGGTGCAAGAC-3′ and LSAMP reverse: 5′- GAATCTAGATTAACATTTGCTGAGAAGGCAGAG-3′. The following primers were used to generate IgLON shRNAmir lentivirus constructs: NTM-A forward: 5′- TGCTGATAGCTTTGGGAAAGGTGGCAGTTTTGGCCACTGACTGACTGCCACCTCCCAAAGCTAT-3′, NTM-A reverse: 5′-CCTGATAGCTTTGGGAGGTGGCAGTCAGTCAGTGGCCAAAACTGCCACCTTTCCCAAAGCTATC-3′, NTM-B forward: 5′- TGCTGCATTCTGGATCTCAATGCTGTGTTTTGGCCACTGACTGACACAGCATTGATCCAGAATG-3′, NTM-B reverse: 5′- CCTGCATTCTGGATCAATGCTGTGTCAGTCAGTGGCCAAAACACAGCATTGAGATCCAGAATGC-3′, LSAMP-A forward: 5′- TGCTGTCTTCTACCACACACCTGAGGGTTTTGGCCACTGACTGACCCTCAGGTGTGGTAGAAGA -3′, LSAMP-A reverse: 5′- CCTGTCTTCTACCACACCTGAGGGTCAGTCAGTGGCCAAAACCCTCAGGTGTGTGGTAGAAGAC -3′, LSAMP-B forward: 5′- TGCTGTTTGCCTGACTGTTCCCTGGTGTTTTGGCCACTGACTGACACCAGGGAAGTCAGGCAAA -3′, and LSAMP-B reverse: 5′- CCTGTTTGCCTGACTTCCCTGGTGTCAGTCAGTGGCCAAAACACCAGGGAACAGTCAGGCAAAC -3′. Preparation of recombinant HSV and lentivirus were performed as previously described^31^. Briefly, IgLON constructs were transiently transfected into 2-2 cells and HEK293T cells for HSV and lentivirus production, respectively. For HSV virus, lipofected cells were infected with a helper virus and harvested by osmotic lysis, followed by three cycles of freeze and thaw using a dry ice/ethanol bath and a 37 °C water bath. For lentivirus production, lipofected cells were incubated for 72 hrs in DMEM supplemented with 10%FBS and the virus was concentrated by centrifugation at 6000 g, overnight at 4 °C.

Recombinant IgLON production - Generation of IgLON constructs have been previously described^14^. Briefly, IgLON constructs were transiently transfected into HEK293T cells^32^. Transfected cells were incubated in the serum-free medium, OptiMEM (Gibco), and the media were collected 4 days post-transfection. IgLON-Fc proteins were purified by affinity chromatography with protein A Sepharose beads.

### DRG neurons

Embryonic day 18-19 (E18-19) and postnatal day 4-6 (P4-6) rat DRG neurons were dissected in ice-cold Leibovitz (L-15) medium (Gibco). DRG tissue was dissociated in 0.25%Trypsin-EDTA (Invitrogen) and gently triturated. DRG were seeded in culture plates pre-coated with Poly-L-Lysine (100 μg/mL, Sigma-Aldrich). Culture medium consisted of Neurobasal (Gibco), 1%B27 (Gibco), 50 μg/mL penicillin-streptomycin (Gibco), 2 mM L-glutamine (Gibco) and 50 nM NGF.

### Outgrowth assays

DRG neurons (20000 neurons/cm^2^) were seeded in a 96-well plate previously coated with PLL. After 2 hrs, the medium was replaced with culture medium including pan-metalloproteinase inhibitors, Batimastat (BB-94; 5 μM, Tocris), Ilomastat (GM6001; 20 μM, CALBIOCHEM), Ilomastat-negative control (GM-I; 20 μM, CALBIOCHEM), ADAM10 inhibitor (GI254023X, 5 μM, Tocris), MMP9 inhibitor (MMP9I, 5 nM, Millipore) or TIMP1, TIMP2, TIMP3 (20 μg/mL, R&D systems) for 24 hrs. For IgLON overexpression, DRG neurons were infected 2 hrs after plating at an MOI 1 for 6 hrs. For NTM land LSAMP loss-of-function, DRG neurons at 3DIV were infected with corresponding lentivirus at an MOI 10 for 4 hrs. After 8DIV, embryonic DRG neurons were reseeded on PLL coated 96-well plate for outgrowth assays. For postnatal stages, DRG neurons were cultured for 8 days on PLL and laminin (10 μg/mL) coated plates and reseeded on PLL and laminin (1 μg/mL). Outgrowth assays with immobilized recombinant IgLON proteins were previously described^14^. Briefly, IgLON-Fc proteins in PBS solution were coated in a 96-well plate for 3 hours. DRG neurons were seeded in IgLON-Fc coated wells, fixed and immunostained after 24 hours. DRG neurons were fixed in 4% PFA, 20% sucrose in PBS for 30 min. Neurons were blocked in 5%BSA and 0.2%Triton X-100 in PBS solution for 1 hour and stained for βIII-tubulin antibody and Hoechst 33342 stain (Sigma-Aldrich). Fluorescent images were automatically acquired and analyzed through the MetaXpress software on an ImageXpress system to obtain the total outgrowth per neuron quantification. The BIII tubulin signal in micrographs presented in the manuscript has been saturated to present a complete morphological trace of the neurons.

### Apoptosis assay

Apoptosis was detected as previously described using a commercially available *In Situ* Cell Death Detection Kit (Roche), following the manufacturer’s instructions. DRG neurons were counterstained with anti-βIII-tubulin and Hoechst 33342 and imaged using the ImageXpress system microscope. The percentage of TUNEL positive neurons was determined using the MultiWavelength Cell Scoring module of MetaXpress.

### RT-PCR

RNA was isolated from dissociated embryonic and postnatal DRGs cultures, followed by a reverse transcriptase polymerase chain reaction (RT-PCR). Rat IgLON primers have been previously described^14^.

### Immunochemistry

Shedding experiments were conducted on DRG membrane extracts and dissociated embryonic DRG neurons. For membrane extracts, dissected tissue was homogenized in ice-cold homogenizing solution (1 mM NaHCO_3_, 0.2 mM CaCl_2_, 0.2 mM MgCl_2_ at pH7) and centrifuged to dispose of any residual tissue. Supernatants were collected and centrifuged for 45 min at 25000 g. The pellet was resuspended in Neurobasal medium supplemented with DMSO, PI-PLC and pan-metalloproteinase inhibitor (BB-94). After 5 hrs, the medium was centrifuged for 1 hr at 100000 g. For dissociated neurons, neuronal cultures were infected at 3DIV. After two days, media was changed for Neurobasal media enriched with DMSO, MMP9 inhibitor, ADAM10 inhibitor or PI-PLC. Supernatants were collected, concentrated and analyzed by western blotting.

### Statistics

For multiple comparisons, 1-way ANOVA followed by Bonferroni post-hoc test were performed using GraphPad Prism software.

### Data Availability Statement

All data generated or analyzed during this study are included in this published article.

## Electronic supplementary material

Supplementary Data
