# Supplementary material for: Ectodomain shedding of Limbic System-Associated Membrane Protein (LSAMP) by ADAM Metallopeptidases promotes neurite outgrowth in DRG neurons
Source: Sci Rep. 2017 Aug 11;7:7961. doi: 10.1038/s41598-017-08315-0 (PMC5554145; doi:10.1038/s41598-017-08315-0)
Supplement: Supplementary file 1 — Supplementary Data [file 41598_2017_8315_MOESM1_ESM.pdf]

Supplementary Data

Ectodomain shedding of Limbic System-Associated Membrane Protein (*LSAMP) by ADAM Metallopeptidases promotes neurite outgrowth in DRG neurons*

Ricardo L. Sanz[1], Gino B. Ferraro[1], Marie-Pier Girouard[1] and Alyson E. Fournier[1*]

[1]Montreal Neurological Institute, Department of Neurology and Neurosurgery, Montreal, H3A 2B4, Canada

*Address correspondence to: Alyson Fournier, Montréal Neurological Institute, BT-109, 3801, Rue University, Montréal, QC, H3A 2B4. alyson.fournier@mcgill.ca

Supplementary Figure Legends

Supplementary Figure S1. Full length Western blots for Fig. 4. A. Blots for Fig. 4b. B. Blots for Fig. 4C. C, D. Blots for 4D. E. Blots for 4E.

Supplementary Figure S2. Full length Western blots for Fig. 6. A. Blots for Fig. 6a.

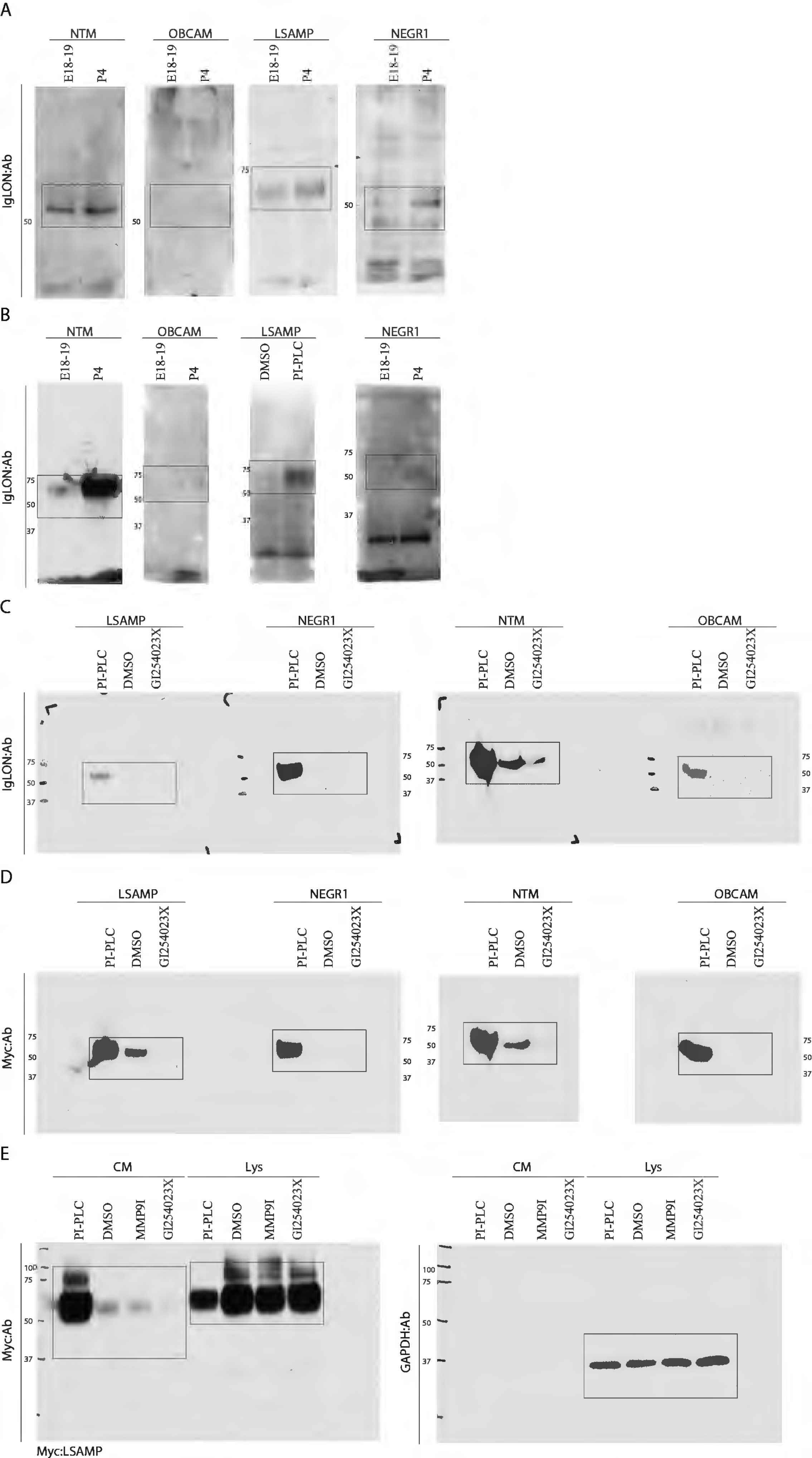
A
NTM
OBCAM
LSAMP
NEGR1
E18-19
P4
IgLON:Ab
B
DMSO
PI-PLC
C
GI254023X
D
Myc:Ab
E
CM
Lys
MMP9I
GAPDH:Ab
Myc:LSAMP

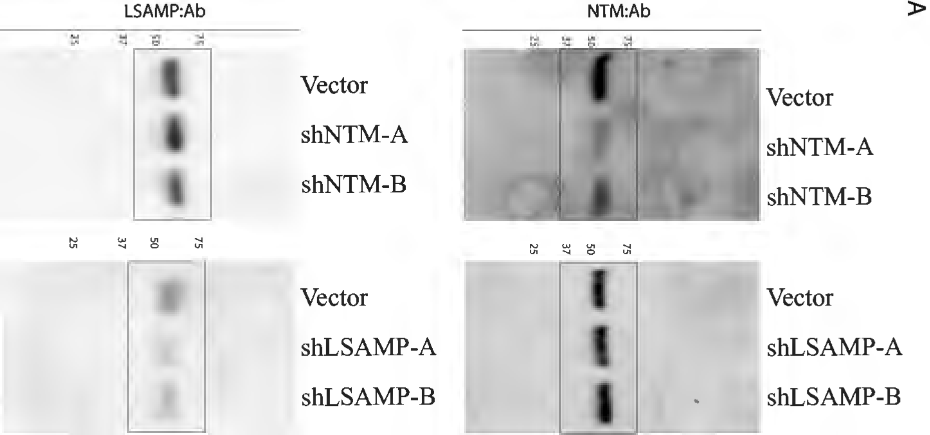
A
LSAMP:Ab
25
37
50
75
Vector
shNTM-A
shNTM-B
25
37
50
75
Vector
shLSAMP-A
shLSAMP-B
NTM:Ab
25
37
50
75
Vector
shNTM-A
shNTM-B
25
37
50
75
Vector
shLSAMP-A
shLSAMP-B
